# Role of α2δ-3 in regulating calcium channel localization at presynaptic active zones during homeostatic plasticity

**DOI:** 10.3389/fnmol.2023.1253669

**Published:** 2023-11-07

**Authors:** Yanfeng Zhang, Ting Wang, Yimei Cai, Tao Cui, Michelle Kuah, Stefano Vicini, Tingting Wang

**Affiliations:** ^1^Department of Pediatric Neurology, First Hospital of Jilin University, Changchun, Jilin, China; ^2^Department of Pharmacology and Physiology, Georgetown University Medical Center, Washington, DC, United States; ^3^Interdisciplinary Program in Neuroscience, Georgetown University Medical Center, Washington, DC, United States

**Keywords:** α2δ-3, voltage gated calcium channel, presynaptic homeostatic plasticity, neurotransmitter release, trafficking, autism, epilepsy, gabapentin

## Abstract

The homeostatic modulation of synaptic transmission is an evolutionarily conserved mechanism that is critical for stabilizing the nervous system. At the *Drosophila* neuromuscular junction (NMJ), presynaptic homeostatic potentiation (PHP) compensates for impairments in postsynaptic glutamate receptors due to pharmacological blockade or genetic deletion. During PHP, there is an increase in presynaptic neurotransmitter release, counteracting postsynaptic changes and restoring excitation to baseline levels. Previous studies have shown that α2δ-3, an auxiliary subunit of voltage-gated calcium channels (VGCCs), is essential for both the rapid induction and sustained expression of PHP at the *Drosophila* NMJ. However, the molecular mechanisms by which α2δ-3 regulates neurotransmitter release during PHP remain to be elucidated. In this study, we utilized electrophysiological, confocal imaging, and super-resolution imaging approaches to explore how α2δ-3 regulates synaptic transmission during PHP. Our findings suggest that α2δ-3 governs PHP by controlling the localization of the calcium channel pore-forming α1 subunit at presynaptic release sites, or active zones. Moreover, we examined the role of two structural domains within α2δ-3 in regulating neurotransmitter release and calcium channel localization. Our results highlight that these domains in α2δ-3 serve distinct functions in controlling synaptic transmission and presynaptic calcium channel abundance, at baseline in the absence of perturbations and during PHP. In summary, our research offers compelling evidence that α2δ-3 is an indispensable signaling component for controlling calcium channel trafficking and stabilization in homeostatic plasticity.

## 1. Introduction

Homeostatic regulation acts as a protective mechanism, operating on synaptic, cellular, and circuit levels to stabilize brain function in the presence of perturbations (Marder and Goaillard, 2006; Turrigiano, 2008; Davis and Muller, 2015). Impairments in homeostatic regulation are closely associated with synaptic and neural network instability, which could underpin the central symptoms of chronic neurological disorders such as epilepsy, Autism Spectrum Disorder (ASD), and neurodegeneration (Ramocki and Zoghbi, 2008; Dickman and Davis, 2009; Wondolowski and Dickman, 2013; Jang and Chung, 2016; Styr and Slutsky, 2018; Orr et al., 2020). Presynaptic homeostatic potentiation (PHP) is a form of homeostatic modulation of synaptic transmission. This robust and dynamic process compensates for diminished postsynaptic receptor sensitivity by increasing presynaptic neurotransmitter release, thereby stabilizing postsynaptic excitation (Davis and Muller, 2015; Frank et al., 2020). PHP can be rapidly induced within minutes through the pharmacological inhibition of glutamate receptors and can be sustained chronically for months (Petersen et al., 1997; Frank et al., 2006). This phenomenon, evolutionarily conserved from *Drosophila* to humans, plays a vital role in stabilizing synaptic function in both the central and peripheral nervous systems (Cull-Candy et al., 1980; Plomp et al., 1992; Dickman and Davis, 2009; Delvendahl et al., 2019; Chipman et al., 2022). Dysregulation of various disease-related molecules, such as *Dysbindin* (associated with Schizophrenia; Dickman and Davis, 2009), *Rim* [associated with ASD (Muller et al., 2012; Wang et al., 2016)], calcium channel pore-forming α1 subunit [associated with epilepsy (Muller and Davis, 2012)], and Amyloid β [associated with Alzheimer’s Disease (Cai et al., 2023; Yin et al., 2023)] leads to a complete disruption of PHP in *Drosophila*, underscoring the potential role of PHP in maintaining nervous system stability and the development of neurological disorders.

Voltage-gated calcium channels (VGCCs) play a crucial role in mediating calcium influx into presynaptic terminals, thus triggering neurotransmitter release at presynaptic active zones (Nanou and Catterall, 2018; Dolphin and Lee, 2020). The abundance, organization, and coupling of presynaptic calcium channels with synaptic vesicles are critical factors that determine release probability and impact synaptic strength (Dittman and Ryan, 2019; Cunningham and Littleton, 2022). Previous studies have demonstrated that two cellular processes are essential for presynaptic homeostatic potentiation: a compensatory increase in presynaptic calcium influx and an expansion of the readily releasable vesicle pool (RRP; Zhao et al., 2011; Muller et al., 2012; Muller and Davis, 2012). However, the exact molecular mechanisms that coherently regulate presynaptic calcium influx and RRP size, ultimately governing neurotransmitter release during PHP, remain largely undefined. Recent advancements in confocal and super-resolution imaging have unveiled dynamic modulations in the abundance and organization of various active zone components and calcium channels, following the inhibition of postsynaptic glutamate receptors in both *Drosophila* and mammalian systems (Tang et al., 2016; Bohme et al., 2019; Gratz et al., 2019; Ghelani et al., 2023). These observations underscore that the trafficking and stabilization of presynaptic calcium channels are vital aspects of homeostatic plasticity.

VGCCs consist of a pore-forming α1 subunit that dictates calcium influx through the channel and auxiliary subunits that modulate the trafficking and biophysical properties of the calcium channel (Dolphin, 2021). The α2δ proteins belong to a family of auxiliary subunits of VGCCs. They undergo post-translational processing, resulting in an extracellular, glycosylated α2 domain linked to a GPI-anchored δ domain through a disulfide bond (De Jongh et al., 1990; Davies et al., 2010). The α2δ proteins interact with the pore-forming α1 subunit of calcium channels in the endoplasmic reticulum (ER) during early secretory trafficking, playing a crucial role in controlling the surface expression of VGCCs (Canti et al., 2005; Hoppa et al., 2012; Chen et al., 2023a). Abnormal functionality of α2δ is broadly associated with various neurological disorders, including epilepsy, neuropathic pain, and ASD (Barclay et al., 2001; Neely et al., 2010; De Rubeis et al., 2014; D'Arco et al., 2015). Among the α2δ subunits, α2δ-1 and α2δ-2 are direct binding targets of gabapentin and pregabalin, two anti-epileptic and anti-hyperalgesic drugs (Hendrich et al., 2008; Patel et al., 2018). In *Drosophila*, *α2δ-3*, a homolog of mammalian *α2δ-3*, has been shown to be necessary for normal synaptic morphogenesis and synaptic transmission (Dickman et al., 2008; Ly et al., 2008; Kurshan et al., 2009; Hoover et al., 2019). In previous studies, we have identified *α2δ-3* as a crucial gene for PHP in an electrophysiology-based genetic screen in *Drosophila*. We established that *α2δ-3* is necessary for the compensatory increase in presynaptic calcium influx and neurotransmitter release during PHP (Wang et al., 2016). However, the specific mechanisms that govern *α2δ-3*-mediated regulation of calcium influx during PHP are yet to be elucidated.

In our quest to understand if α2δ-3 controls neurotransmitter release by regulating the trafficking and localization of presynaptic calcium channels during homeostatic plasticity, we focused on two critical structural domains within α2δ that have been linked to VGCC trafficking. The von Willebrand Factor A (vWA) domain in α2δ has been implicated in the trafficking and plasma membrane association of the α1 pore-forming subunit of the calcium channels (Canti et al., 2005; Hoppa et al., 2012; Cassidy et al., 2014; Chen et al., 2023a). Additionally, gabapentin’s interaction with a triple arginine site (arginine-arginine-arginine, RRR) in α2δ-1 and α2δ-2 influences the recycling of calcium channels (Field et al., 2006; Tran-Van-Minh and Dolphin, 2010; Cassidy et al., 2014). To probe the role of α2δ-3 in VGCC trafficking during PHP, we introduced loss-of-function mutations into two specific sites: the Metal Ion-Dependent Adhesion Site (MIDAS) in the vWA domain, and the arginine site (arginine-leucine-arginine, RLR) in *Drosophila* α2δ-3. Using a combination of electrophysiology, confocal imaging, and STED super-resolution imaging techniques, we derived several crucial insights. First, we provided evidence suggesting that the MIDAS motif within the vWA domain controls both baseline synaptic transmission and PHP by regulating the accumulation of presynaptic calcium channels at active zones. Conversely, the RLR site in α2δ-3 specifically regulates synaptic transmission and calcium channel abundance at baseline, but is not necessary for PHP or the PHP-dependent regulation of presynaptic calcium channels. These findings strongly suggest that α2δ-3 is required for neurotransmitter release during PHP by controlling presynaptic calcium channel trafficking. Furthermore, the differentiated roles of the distinct α2δ-3 domains in regulating baseline neurotransmitter release and PHP underscore the unique signaling properties of α2δ-3 in calcium channel trafficking during homeostatic plasticity.

## 2. Materials and methods

### 2.1. *Drosophila* stocks and husbandry

The *w^1118^* strain was used as a *wild-type* (*wt*) control, unless otherwise noted. *Drosophila* alleles used were raised at 25°C, unless otherwise noted in Figure Legends, and all flies were raised on standard molasses food. The following *Drosophila* stocks were used: *GluRIIA^sp16^*, *Elav^C155^-Gal4* (BDSC BL458), and *OK371-Gal4* (BDSC BL26160). The *α2δ-3^k10814^* and *α2δ-3^106^* alleles were generously provided by Dr. Yuh-Nung Jan (University of California, San Francisco; Howard Hughes Medical Institute) and *Cac^sfGFP^* was a generous gift from Dr. Kate O’Connor-Giles (Brown University). Flies bearing *UAS-stj-3HA* (F001252) were obtained from FlyORF (University of Zurich).

### 2.2. Generation of *Drosophila* transgenic lines

The *α2δ-3* coding sequence was cloned from *UAS-stj-3HA* (FlyORF F001252) transgenic flies and inserted into the pENTR-D-TOPO vector using the pENTR Directional TOPO Cloning Kit (Invitrogen K240020). Mutations were introduced in the MIDAS motif (**D**G**S**G**S** to **A**G**A**G**A**, *UAS-α2δ-3^DSS-AAA^*) or RLR site (**RLR** to **AAA**, *UAS-α2δ-3^RLR-AAA^*) of α2δ-3 using the Q5 site-directed mutagenesis kit (NEB E0554S). For generation of final constructs, pENTR vectors were recombined with destination vector pUASg-HA_attB (DGRC 1423) using LR clonase II enzyme (Invitrogen 11791020). Once final constructs were generated, they were sent to BestGene Inc. (Chino Hill, CA) for injection, using ZH-86Fb (III) as the targeted insertion strain. *UAS-α2δ-3^DSS-AAA^-3HA* and *UAS-α2δ-3^DSS-AAA^-3HA* transgenic lines were validated through PCR and sequencing, and these lines were subsequently used in experiments.

### 2.3. Protein structure prediction and alignment

**Figure 1:**
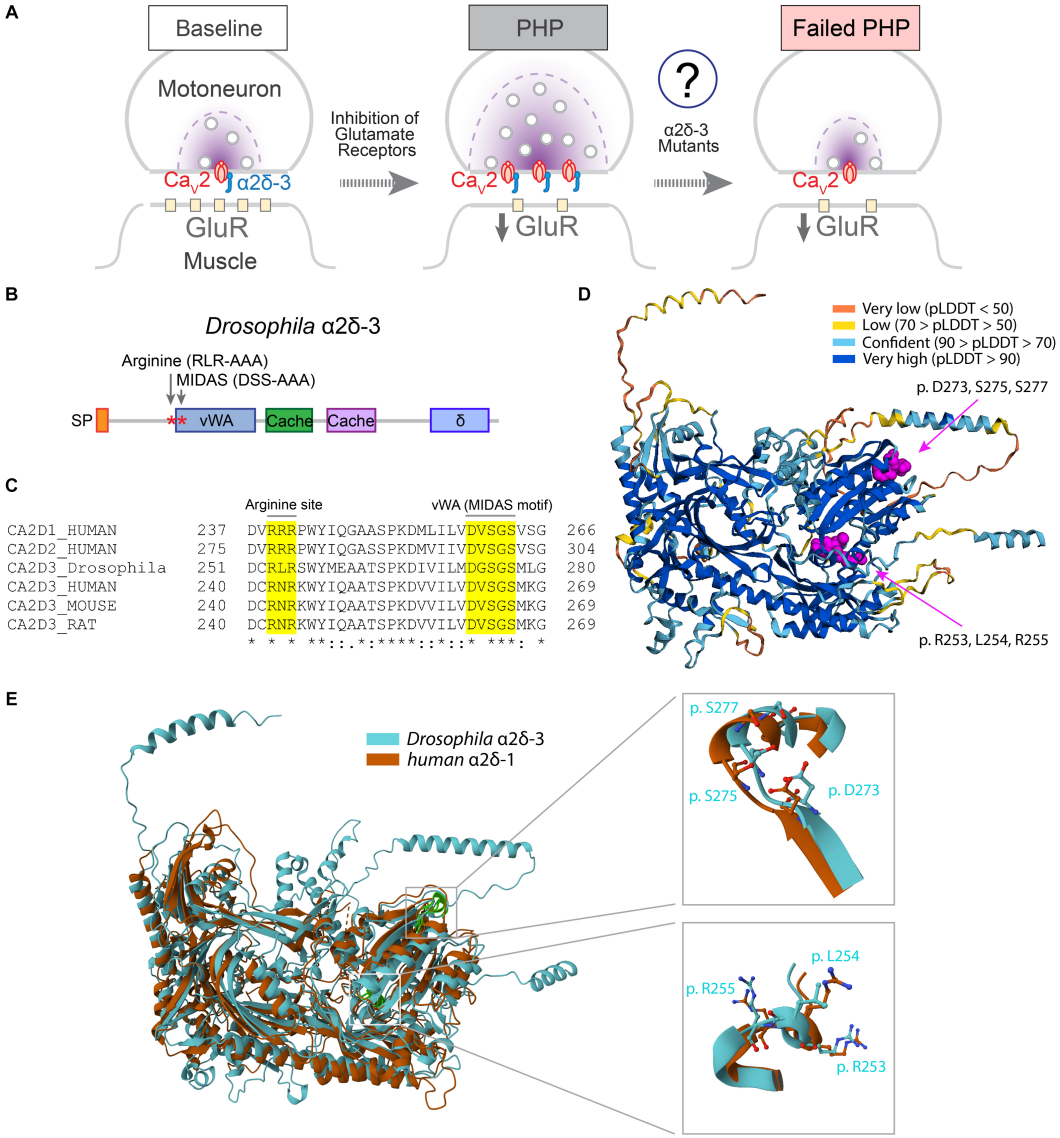
Protein domains required for VGCC trafficking are conserved in α2δ-3. **(A)** Schematic to show PHP at the *Drosophila* NMJ. Inhibition of postsynaptic glutamate receptor sensitivity leads to a calcium channel-dependent increase of presynaptic release in motoneurons. The function of α2δ-3 in regulating calcium channel localization at presynaptic active zones during PHP remains to be elucidated. **(B)** Schematic to show protein structural domains and mutations introduced into *Drosophila* α2δ-3. Signal peptide (SP), von Willebrand Factor A(vWA), Cache (Cache), and δ domains are shown. **(C)** Alignment of α2δ-3 protein sequences across different species. Mutated amino acids in the MIDAS motif in vWA domain (**D**G**S**G**S** to **A**G**A**G**A**, *UAS-α2δ-3^DSS-AAA^*) and RLR site (**RLR** to **AAA**, *UAS-α2δ-3^RLR-AAA^*) are highlighted. **(D)** The structure of *Drosophila* α2δ-3 predicted by AlphaFold. Prediction confidence levels (pLDDT) are color-labeled. The MIDAS motif and RLR site are shown as spheres with carbon atoms colored in magenta. **(E)** Superimposed Cryo-EM structure of human α2δ-1 (orange) and AlphaFold-predicted structure of *Drosophila* α2δ-3 (blue). Comparisons of the MIDAS motif (upper right) and RLR site (lower right) are shown. Individual residues are shown in stick presentation.

**Figure 2:**
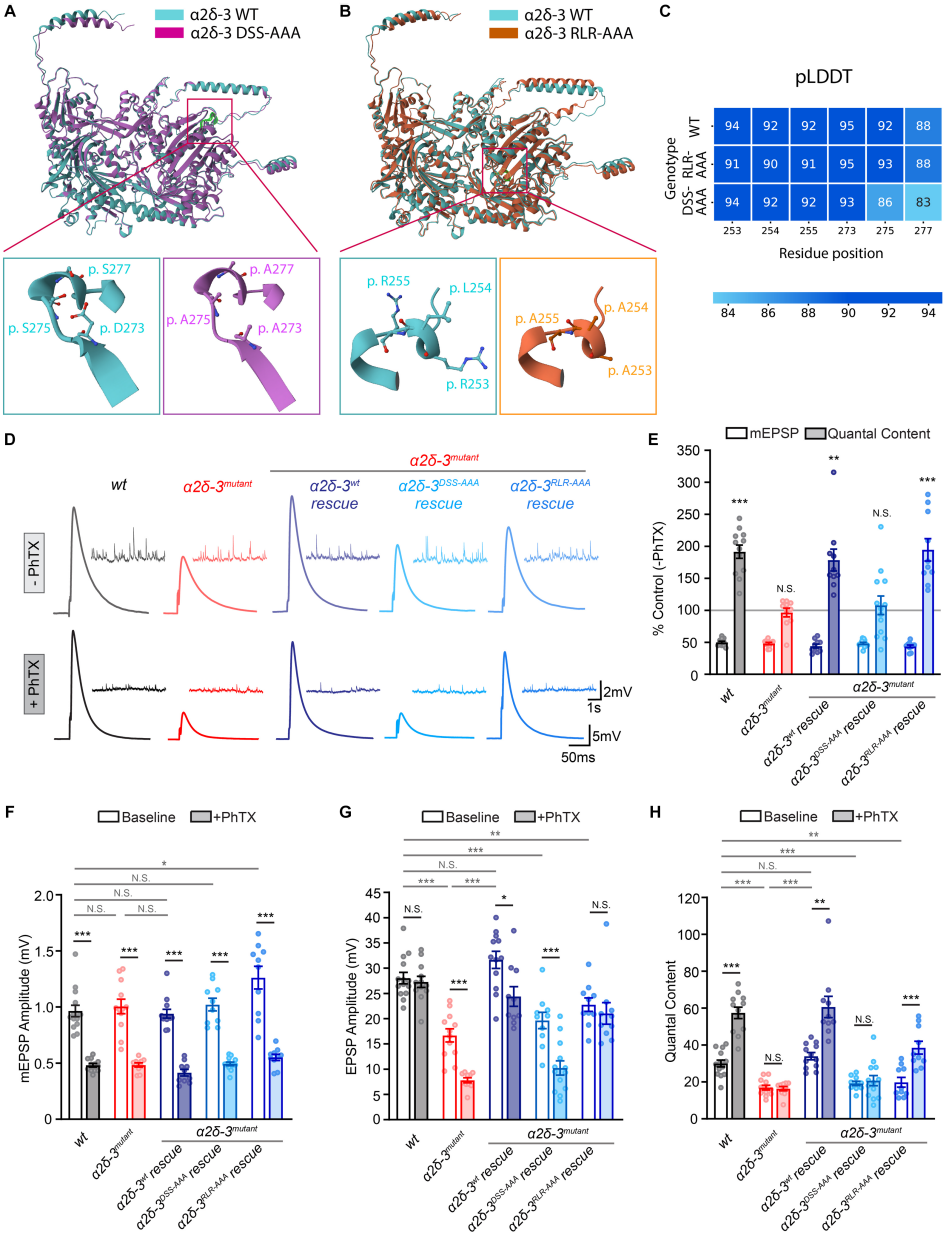
The MIDAS motif and RLR site in α2δ-3 display distinct functions in regulating basal synaptic transmission and acute PHP. **(A–C)** *Drosophila* α2δ-3 protein sequence and effects of mutations in the MIDAS motif **(A)** and RLR site **(B)** predicted by AlphaFold, and the prediction confidence (pLDDT, **C**) for individual residues (DSS-AAA: p.273, 275, 277; RLR-AAA: p.253, 254, 255). Mutated amino-acid residues in the MIDAS motif (magenta, **A**) and RLR site (orange, **B**) are compared to the *wild-type* (blue) as stick illustrations in lower panels. **(D)** Representative mEPSP and EPSP traces in *wild-type* (*wt*), the *α2δ-3* homozygous mutant (*α2δ-3^mutant^*, *α2δ-3^k10814^/α2δ-3^106^*), pan-neuronal overexpression of *α2δ-3^wt^* (*α2δ-3^wt^* rescue, *Elav^c155^-Gal4;α2δ-3^k10814^/α2δ-3^106^;UAS-α2δ-3^wt^*), pan-neuronal overexpression of *α2δ-3^DSS-AAA^* (*α2δ-3^DSS-AAA^* rescue, *Elav^c155^-Gal4;α2δ-3^k10814^/α2δ-3^106^;UAS-α2δ-3^DSS-AAA^*), and pan-neuronal overexpression of *α2δ-3^RLR-AAA^* (*α2δ-3^RLR-AAA^* rescue, *Elav^c155^-Gal4;α2δ-3^k10814^/α2δ-3^106^;UAS-α2δ-3^RLR-AAA^*) in the *α2δ-3* homozygous mutant background, in the absence (−PhTX) and presence of philanthotoxin (+PhTX). **(E)** Normalized average mEPSP amplitude (open bars) and presynaptic release (quantal content, QC; filled bars) are presented as the percentage of change in the presence of PhTX compared to the same genotype recorded in the absence of PhTX. Genotypes and sample sizes: *wild-type* (*wt*, *n* = 14 −PhTX, 12 +PhTX), the *α2δ-3* homozygous mutant (*α2δ-3^mutant^*, *α2δ-3^k10814^/α2δ-3^106^*, *n* = 12 −PhTX, 10 +PhTX), pan-neuronal overexpression of *α2δ-3^wt^* (*α2δ-3^wt^* rescue, *Elav^c155^-Gal4;α2δ-3^k10814^/α2δ-3^106^;UAS-α2δ-3^wt^*, *n* = 12 −PhTX, 10 +PhTX), pan-neuronal overexpression of *α2δ-3^DSS-AAA^* (*α2δ-3^DSS-AAA^* rescue, *Elav^c155^-Gal4;α2δ-3^k10814^/α2δ-3^106^;UAS-α2δ-3^DSS-AAA^*, *n* = 10 −PhTX, 13 +PhTX), and in pan-neuronal overexpression of *α2δ-3^RLR-AAA^* (*α2δ-3^RLR-AAA^* rescue, *Elav^c155^-Gal4;α2δ-3^k10814^/α2δ-3^106^;UAS-α2δ-3^RLR-AAA^*, *n* = 10 −PhTX, 10 +PhTX) in the *α2δ-3* homozygous mutant background. Mean ± SEM; *q < 0.05, **q < 0.01, ***q < 0.001, N.S. not significant; Brown-Forsythe and Welch ANOVA (non-equal variance) with Benjamini and Hochberg FDR method was used to correct for multiple comparisons. Non-normalized raw data are used for statistical tests. **(F–H)** Non-normalized raw data for average mEPSP amplitude **(F)**, EPSP amplitude **(G)**, and presynaptic release (quantal content, **H**) in the absence (baseline, open) and presence (+PhTX, filled bars) of PhTX. Genotypes and sample sizes are as presented in **(E)**. Mean ± SEM; *q < 0.05, **q < 0.01, ***q < 0.001, N.S. not significant; Brown-Forsythe and Welch ANOVA (non-equal variance) with Benjamini and Hochberg FDR method was used to correct for multiple comparisons.

The protein sequences used for alignment were as follows: human CA2D1 (P54289), CA2D2 (Q9NY47), CA2D3 (Q8IZS8), mouse CA2D3 (Q9Z1L5), rat CA2D3 (Q8CFG5), and *Drosophila* CA2D3 (Q7K0H4, isoform B). The alignment was performed using CLUSTAL O (1.2.4). To generate predicted structures for *Drosophila wild-type* CA2D3 and CA2D3 with DSS-AAA or RLR-AAA mutations, we utilized ColabFold (Mirdita et al., 2022), which combines the fast homology search of MMseqs2 (Many-against-Many sequence searching) with AlphaFold2 (Jumper et al., 2021). For alignment in Figure 1E, we used the cryo-EM structure of human CA2D1 (pdb8FD7; Chen et al., 2023a) and *wild-type Drosophila* CA2D3 structure predicted by AlphaFold2. The alignment and presentation of *wild-type* and mutated *Drosophila* CA2D3 structures were performed using RCSB PDB in Figures 2A,B. The protein structures of *wild-type* and mutated *Drosophila* CA2D3, custom Python codes for AlphaFold protein structure presentations, and AlphaFold pLDDT statistics are available at: https://github.com/wanglab-georgetown/alphafold_a2d3.

### 2.4. Electrophysiology

Sharp-electrode recordings were made from muscle 6 at abdominal segments 2 and 3 of male and female 3^rd^ instar larvae using an Axoclamp 900A amplifier (Molecular Devices) as previously described (Frank et al., 2006). For *α2δ-3* rescue experiments using *Elav^C155^-Gal4* (located on the X chromosome), only male larvae were included in recordings. HL3 saline solution containing the following concentrations (in mM) was used for all current clamp recordings: 70 NaCl, 5 KCl, 10 MgCl_2_, 10 NaHCO_3_, 115 Sucrose, 5 Trehalose, 5 HEPES, and 0.3 CaCl_2_. EPSP and mEPSP traces were analyzed using StimFit (0.15.8) and MiniAnalysis (6.0.3 Synaptosoft). To rapidly induce PHP, larvae were incubated in 20 μM Philanthotoxin-433 (PhTX, Santa Cruz Biotechnology 276684-27-6 or AOBIOUS AOB0876) in an un-stretched, partially dissected preparation for 10 min, following the method described in Frank et al. (2006). For each NMJ, the average amplitudes of evoked EPSPs are based on the mean peak amplitudes in response to 20–30 individual stimuli. mEPSPs were recorded continuously for 60–90 s. Quantal content was estimated for each NMJ as the ratio of EPSP amplitude/mEPSP amplitude. The mean value across all NMJ for a given genotype is reported.

### 2.5. Immunohistochemistry

Immunohistochemistry (IHC) was conducted using standard protocols as previously described (Wang et al., 2014, 2020). Briefly, dissected third instar larvae were fixed in ice-cold ethanol for 5 min on ice, followed by rinses in PBST (PBS with 0.01% Triton X-100) for 6 times, 10 min each at room temperature. The preparations were then incubated in blocking solution (PBST with 5% normal goat serum) for 2 h at room temperature. Rabbit anti-GFP (1:200, Invitrogen G10362) and mouse anti-Bruchpilot (Brp) antibodies (1:200, DSHB nc82) were used for overnight incubation at 4°C with rotation. For confocal imaging, after the primary antibody incubation, the preparations were rinsed for 6 times, 10 min each in PBST. They were then incubated with Alexa 488-conjugated goat anti-rabbit (1:300, Invitrogen A-11008), Alexa Cy3-conjugated goat anti-mouse (1:300, Invitrogen A-10521), and Alexa 647-conjugated goat anti-HRP (1:100, Jackson Immuno Research Laboratories 123-605-021) antibodies. The preparations were mounted with Fisher Brand #1 coverslips using VECTASHIELD antifade mounting medium (H-1000-10). For STED imaging, after the primary antibody incubation, the preparations were incubated with Alexa 594-conjugated goat anti-mouse (1:300, Invitrogen R37121) and ATTO 647 N-conjugated goat anti-rabbit antibodies (1:500, Rockland 611-156-122S) in PBST for 2 h at room temperature. After 6 times, 10 min rinses in PBST, the preparations were mounted with Fisher Brand #1.5 coverslips using Prolong Gold mounting medium (Invitrogen P36930). In experiments to examine calcium channel abundance during acute PHP, a 10 min incubation with 20 μM Philanthotoxin-433 (PhTX, AOBIOUS AOB0876) was performed to induce PHP prior to fixation. For each IHC experiment, the consistency of immunolabeling is maintained by dissecting, fixing and staining preparations of all genotypes simultaneously. This ensures that all preparations were subjected to identical experimental conditions, such as antibody incubation, thereby minimizing variability.

### 2.6. Image acquisition and analysis

Confocal images of the NMJ were acquired using a laser scanning confocal microscope (LSM 880, Carl Zeiss). Z-stacks of the NMJ on muscle 6/7 segments A2 and A3 were captured using a 63x objective (Plan-Apochromat 63x/1.40 Oil DIC M27), and maximum projections were used for analysis in Fiji (NIH). Fluorescence intensities in each channel of the confocal images spanned a dynamic range without reaching saturation. STED images of single 1b boutons (three most distal boutons per synapse) were obtained from the NMJ on muscle 6/7 segment A2 using a Nikon Eclipse Ti2-E confocal microscope equipped with an Abberior STEDYCON unit and a Nikon Plan Apo 100× NA1.45 Lambda Oil DIC N2 objective. The Alexa 594 and ATTO 647 N channels were scanned using 561 nm (excitation)/775 nm (STED) and 640 nm (excitation)/775 nm (STED) lasers, respectively. This setup provides a resolution of 40-60 nm on the X/Y plane. Each Z-stack consisted of 4 sections with a 0.5 μm step size to minimize bleaching. All images were captured in 16-bit format with a size of 8 μm × 8 μm. To maintain consistency of confocal and STED imaging, samples from each batch of IHC experiments were imaged using identical settings, and all within the same day. Maximum projections were used for analysis in Fiji (NIH) and presentation in Figures.

All imaging analyses were executed on unsaturated raw data. For confocal data analysis, binary masks were generated by applying a threshold to the Brp (cy3) channel, which were then transferred to the Cac (488) channel to quantify the mean and integrated intensity of Cac at individual active zones (inside Brp puncta) for each synapse. For STED data analysis, Z-stacks of boutons were processed using Fiji (NIH). After splitting the channels and creating maximum-intensity projections, a threshold was applied to each channel to isolate the active zones (Alexa 594 channel) or Cac (ATTO 647 N channel). The threshold for each channel remained consistent for all images collected in one experimental repeat. Particle analysis was performed to quantify intensity and area. The mean intensity (average fluorescence), integrated intensity (sum fluorescence), and area of each particle were then measured and reported for each bouton (STED).

### 2.7. Statistics

The data are presented as Mean ± Standard Error of the Mean (SEM), with the precise sample sizes indicated in the Figure Legends. Statistical analysis was performed using Prism (9.5.1, GraphPad). First, we assessed the normality of residuals from ANOVA tests across all datasets using the D’Agostino-Pearson test. Then, for datasets with normal residuals, we used Brown-Forsythe and Welch ANOVA (non-equal variance) tests. For datasets with non-normal ANOVA residuals, the nonparametric Kruskal-Wallis test was applied. In case of comparing more than two conditions, we used the original False Discovery Rate (FDR) method of Benjamini and Hochberg to correct for multiple comparisons. The q-values obtained from the FDR correction are reported in the Figure Legends.

## 3. Results

### 3.1. Essential protein domains for VGCC trafficking are conserved in α2δ-3

The α2δ proteins are located at the extracellular face of presynaptic release sites and interact directly with the α1 subunit of VGCCs (Figure 1A). In mammalian α2δ-1 and α2δ-2, the vWA domain and the arginine-arginine-arginine (RRR) gabapentin-binding site are essential for calcium channel trafficking (Canti et al., 2005; Field et al., 2006; Hoppa et al., 2012). To investigate the domains involved in regulating calcium channel trafficking in α2δ-3, we aligned the protein sequences and identified conserved regions (Figures 1B,C). The MIDAS motif within the vWA domain of α2δ-1 and α2δ-2 is crucial for proper calcium channel distribution and synaptic transmission (Hoppa et al., 2012). We observed that the MIDAS motif (DxSxS) in α2δ proteins is highly conserved across different species (Figure 1C). Furthermore, gabapentin and pregabalin bind directly to the RRR site in α2δ-1 and α2δ-2, leading to a decrease in surface expression of calcium channels (Field et al., 2006; Hendrich et al., 2008). In α2δ-3, the first and third arginine residues are conserved, but instead of an RRR site like α2δ-1 and α2δ-2, mammalian α2δ-3 has an arginine-asparagine-arginine (RNR) sequence, while *Drosophila* α2δ-3 has an arginine-leucine-arginine (RLR) sequence (Figure 1C). Based on previous functional studies and the cryo-EM structure of mammalian α2δ-1 (Chen et al., 2023b), we hypothesize that the RLR site in *Drosophila* α2δ-3 may be involved in the regulation of calcium channel trafficking.

The structure of *Drosophila* α2δ-3 protein has not been experimentally determined. Therefore, we used the AlphaFold algorithm (Jumper et al., 2021; Mirdita et al., 2022) to predict the structure of *Drosophila* α2δ-3. The predicted structure of *Drosophila* α2δ-3, particularly the protein domains containing the MIDAS motif and RLR site, showed high confidence according to the pLDDT score (Figure 1D; Tunyasuvunakool et al., 2021). To validate the prediction, we compared the predicted structure of *Drosophila* α2δ-3 with the cryo-EM structure of human α2δ-1 (Ca_v_1.2/Ca_v_β/Ca_v_α2δ-1; Chen et al., 2023a). The alignment between the two structures yielded a template modeling-score (TM-score; Xu and Zhang, 2010) of 0.67 and a root mean square deviation (RMSD; Kufareva and Abagyan, 2012) of 3.05 Å. These results indicate that *Drosophila* α2δ-3 and human α2δ-1 exhibit similar structural characteristics, thereby confirming the accuracy of the AlphaFold prediction (Figure 1E).

### 3.2. Rapid induction of PHP requires the MIDAS motif but not RLR site in α2δ-3

To investigate the functional role of α2δ-3 in regulating calcium channel trafficking during PHP, we generated two mutant forms of α2δ-3 by introducing specific mutations in either the vWA domain or the RLR site. For the vWA domain mutation, we replaced three conserved metal coordinating residues within the MIDAS motif (**D**G**S**G**S**) with alanine residues (**A**G**A**G**A**) in the *UAS-α2δ-3^wt^* transgene, resulting in the *UAS-α2δ-3^DSS-AAA^* mutant (Figures 1B,C, 2A). Additionally, we generated a transgenic line carrying mutations in the RLR site, where the RLR sequence was mutated to AAA, yielding the *UAS-α2δ-3^RLR-AAA^* mutant (Figures 1B,C, 2B).

Using AlphaFold, we predicted the structural impact of these mutations in the MIDAS motif and RLR site. The predicted structures of both the *wild-type* and mutated forms of α2δ-3 showed high confidence for the MIDAS motif and RLR site (Figure 2C). In the DSS-AAA mutant, the negatively charged or polar residues of the MIDAS motif were replaced by alanine residues, which have a shorter carbon backbone and smaller hydrophobic side chains. Similarly, in the RLR-AAA mutant, the positively charged residues of the RLR site were substituted with alanine residues. These mutations may alter the local conformation of the vWA domain and RLR site, thereby disrupting potential interactions with binding partners in the binding pockets (Figures 2A,B). While the exact impact of these mutations requires experimental validation, the overall Alphafold-predicted structure of the α2δ-3 protein was not significantly affected by these mutations, as indicated by the RMSD scores of 0.84Å and 1.45Å when comparing the *wild-type* α2δ-3 to the DSS-AAA and RLR-AAA mutants, respectively.

To assess the effect of mutations in the MIDAS motif and RLR site of α2δ-3 on PHP, we induced PHP by applying the glutamate receptor antagonist Philanthotoxin-433 (PhTX, 20 μM; Frank et al., 2006) and performed electrophysiological recordings at the neuromuscular junction (NMJ). In all genotypes, including the *wild-type*, we observed a reduction of approximately 50% in the average amplitude of miniature excitatory postsynaptic potentials (mEPSPs) upon PhTX application (Figures 2D–F). In the *wild-type* condition, the pharmacological inhibition of postsynaptic glutamate receptors resulted in a significant increase in presynaptic neurotransmitter release, indicated by an increase in quantal content (QC), which compensated for the perturbation and restored the excitatory postsynaptic potential (EPSP) amplitude back to the normal baseline level (Figures 2D,E,G,H). However, in the *α2δ-3* homozygous mutant (*α2δ-3^k10814^/α2δ-3^106^*), when PHP was induced with PhTX, we observed no change in quantal content, and the EPSP amplitude was dramatically reduced, indicating a complete loss of PHP (Figures 2D,E,G,H). These findings are consistent with our previous observations that *α2δ-3* is necessary for the induction of PHP (Wang et al., 2016).

In order to investigate the functional effects of mutations in the MIDAS motif and RLR site of α2δ-3 on PHP, we conducted tissue-specific rescue experiments. Specifically, we selectively overexpressed *wild-type α2δ-3* (*UAS-α2δ-3^wt^*) or *α2δ-3* with mutations in the MIDAS motif (*UAS-α2δ-3^DSS-AAA^*) or RLR site (*UAS-α2δ-3^RLR-AAA^*) in neurons using the *Elav^C155^-Gal4* driver in the background of the *α2δ-3* homozygous mutant (genotypes as indicated in the Figure Legends; Figures 2D–H). Consistent with previous findings, neuronal overexpression of *wild-type α2δ-3* (*α2δ-3^wt^* rescue, *Elav^C155^-Gal4;α2δ-3^k10814^/α2δ-3^106^;UAS-α2δ-3^wt^*) restored the compensatory increase in quantal content in the *α2δ-3* mutant background (Figures 2E,H). Although the EPSP amplitude was not fully restored to the baseline level, there was a significant increase in quantal content when we induced PHP using PhTX in the *α2δ-3^wt^* rescue condition (Figures 2D,E,G,H). These results suggest that *wild-type α2δ-3* expression in neurons rescues the loss of PHP.

Intriguingly, our findings from the neuronal overexpression of *α2δ-3^DSS-AAA^* (*α2δ-3^DSS-AAA^* rescue, *Elav^C155^-Gal4;α2δ-3^k10814^/α2δ-3^106^;UAS-α2δ-3^DSS-AAA^*) and *α2δ-3^RLR-AAA^* (*α2δ-3^RLR-AAA^* rescue, *Elav^C155^-Gal4;α2δ-3^k10814^/α2δ-3^106^;UAS-α2δ-3^RLR-AAA^*) in the *α2δ-3* homozygous mutant background revealed distinct effects on PHP. Specifically, the overexpression of *α2δ-3^DSS-AAA^* failed to rescue the compensatory increase in neurotransmitter release observed during PHP (Figures 2D–H). However, in contrast, the overexpression of *α2δ-3^RLR-AAA^* fully restored PHP (Figures 2D–H). The EPSP amplitude remained significantly reduced in the *α2δ-3^DSS-AAA^* rescue condition in the presence of PhTX (Figure 2G). On the other hand, overexpression of *α2δ-3^RLR-AAA^* precisely restored the EPSP amplitude back to the baseline level after PhTX application (Figure 2G). These results provided compelling evidence that the MIDAS motif, but not the RLR site, in α2δ-3 is crucial for the rapid induction of PHP. The MIDAS motif appears to play a critical role in mediating the compensatory increase in neurotransmitter release during PHP, while the RLR site is not be directly involved in this process.

### 3.3. Basal synaptic transmission requires both the MIDAS motif and RLR site in α2δ-3

We examined the EPSP amplitude and quantal content in the absence of PhTX in the *α2δ-3* homozygous mutant and various rescue conditions. We found that in the *α2δ-3* homozygous mutant (*α2δ-3^k10814^/α2δ-3^106^*), there was a significant reduction in both EPSP amplitude and quantal content, suggesting that *α2δ-3* is crucial for normal neurotransmitter release at baseline (Figures 2D,G,H). As expected, the neuronal-specific overexpression of *α2δ-3^wt^* restored the EPSP amplitude and quantal content in the *α2δ-3* mutant, bringing them back to *wild-type* levels (Figures 2D,G,H). However, in contrast, the neuronal overexpression of either *α2δ-3^DSS-AAA^* or *α2δ-3^RLR-AAA^* did not restore the EPSP amplitude or quantal content in the *α2δ-3* mutant at baseline (Figures 2D,G,H). We observed a slight yet statistically significant increase in mEPSP amplitude in the *α2δ-3^RLR-AAA^* rescue as compared to the *wild-type* control (Figure 2F). This observation suggests potential postsynaptic changes associated with the *α2δ-3^RLR-AAA^* rescue. However, we noted that there was still a dramatic decrease in quantal content in the *α2δ-3^RLR-AAA^* rescue condition compared to the *wild-type* control (Figure 2H). These results highlight that both the MIDAS motif and RLR site in α2δ-3 are necessary for normal presynaptic neurotransmitter release under basal conditions in the absence of PhTX.

In summary, our findings indicate that the MIDAS motif within the vWA domain and RLR site in α2δ-3 play distinct roles in controlling basal synaptic transmission and PHP. The MIDAS motif is essential for both processes, while the RLR site is specifically required for baseline neurotransmitter release and is not involved in PHP. Despite the absence of direct binding between mammalian α2δ-3 and gabapentin (Marais et al., 2001), our study provides evidence that the RLR site in *Drosophila* α2δ-3 is critical for normal synaptic transmission. We hypothesize that the RLR site interacts with endogenous binding partners to regulate the trafficking of VGCCs. These findings shed light on the functional significance of specific protein domains in α2δ-3 and their involvement in the regulation of synaptic plasticity.

### 3.4. Long-term maintenance of PHP requires the MIDAS motif but not the RLR site in α2δ-3

**Figure 3:**
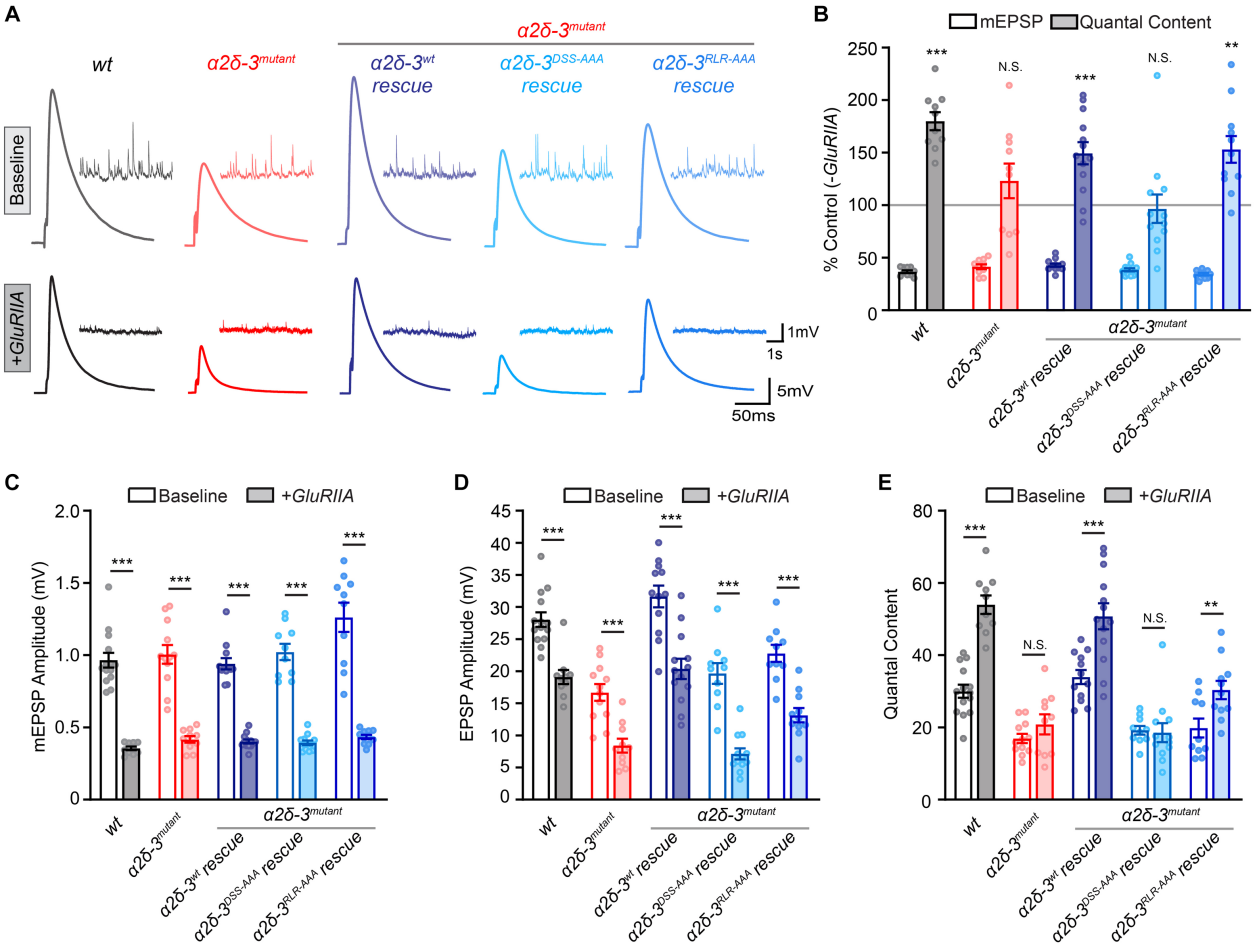
The MIDAS motif, not the RLR site, in α2δ-3 is essential for long-term maintenance of PHP. **(A)** Representative mEPSP and EPSP traces in *wild-type* (*wt*), the *α2δ-3* homozygous mutant (*α2δ-3^mutant^*, *α2δ-3^k10814^/α2δ-3^106^*), pan-neuronal overexpression of *α2δ-3^wt^* (*α2δ-3^wt^* rescue, *Elav^c155^-Gal4;α2δ-3^k10814^/α2δ-3^106^;UAS-α2δ-3^wt^*), pan-neuronal overexpression of *α2δ-3^DSS-AAA^* (*α2δ-3^DSS-AAA^* rescue, *Elav^c155^-Gal4;α2δ-3^k10814^/α2δ-3^106^;UAS-α2δ-3^DSS-AAA^*), and pan-neuronal overexpression of *α2δ-3^RLR-AAA^* (*α2δ-3^RLR-AAA^* rescue, *Elav^c155^-Gal4;α2δ-3^k10814^/α2δ-3^106^;UAS-α2δ-3^RLR-AAA^*) in the *α2δ-3* homozygous mutant background under baseline conditions (baseline, upper panels). Representative mEPSP and EPSP traces in *GluRIIA*, the *GluRIIA,α2δ-3* double homozygous mutant (*GluRIIA,α2δ-3^k10814^/GluRIIA,α2δ-3^106^*), pan-neuronal overexpression of *α2δ-3^wt^* (*α2δ-3^wt^* rescue, *Elav^c155^-Gal4;GluRIIA,α2δ-3^k10814^/GluRIIA,α2δ-3^106^;UAS-α2δ-3^wt^*), pan-neuronal overexpression of *α2δ-3^DSS-AAA^* (*α2δ-3^DSS-AAA^* rescue, *Elav^c155^-Gal4;GluRIIA,α2δ-3^k10814^/GluRIIA,α2δ-3^106^;UAS-α2δ-3^DSS-AAA^*), and pan-neuronal overexpression of *α2δ-3^RLR-AAA^* (*α2δ-3^RLR-AAA^* rescue, *Elav^c155^-Gal4;GluRIIA,α2δ-3^k10814^/GluRIIA,α2δ-3^106^;UAS-α2δ-3^RLR-AAA^*) in the *GluRIIA,α2δ-3* double homozygous mutant background (+*GluRIIA*, lower panels). **(B)** Normalized average mEPSP amplitude (open bars) and presynaptic release (quantal content, QC; filled bars) are presented as the percentage of change in the presence of *GluRIIA* homozygous mutation compared to the same genotype recorded in the absence of *GluRIIA* mutation. Genotypes and sample sizes: *wild-type* (*wt*, *n* = 14 −*GluRIIA*, 10 +*GluRIIA*), the *α2δ-3* homozygous mutant (*α2δ-3^mutant^*, *α2δ-3^k10814^/α2δ-3^106^*, *n* = 12 −*GluRIIA*, 10 +*GluRIIA*), pan-neuronal overexpression of *α2δ-3^wt^* (*α2δ-3^wt^* rescue, *Elav^c155^-Gal4;α2δ-3^k10814^/α2δ-3^106^;UAS-α2δ-3^wt^*, *n* = 12 −*GluRIIA*, 13 +*GluRIIA*), pan-neuronal overexpression of *α2δ-3^DSS-AAA^* (*α2δ-3^DSS-AAA^* rescue, *Elav^c155^-Gal4;α2δ-3^k10814^/α2δ-3^106^;UAS-α2δ-3^DSS-AAA^*, *n* = 10 −*GluRIIA*, 12 +*GluRIIA*), and in pan-neuronal overexpression of *α2δ-3^RLR-AAA^* (*α2δ-3^RLR-AAA^* rescue, *Elav^c155^-Gal4;α2δ-3^k10814^/α2δ-3^106^;UAS-α2δ-3^RLR-AAA^*, *n* = 10 −*GluRIIA*, 11 +*GluRIIA*) in the *α2δ-3* single (−*GluRIIA*) or *GluRIIA,α2δ-3* double (+*GluRIIA*) mutant background. Mean ± SEM; *q < 0.05, **q < 0.01, ***q < 0.001, N.S. not significant; Brown-Forsythe and Welch ANOVA (non-equal variance) with Benjamini and Hochberg FDR method was used to correct for multiple comparisons. Non-normalized raw data are used for statistical tests. **(C–E)** Non-normalized raw data for average mEPSP amplitude **(C)**, EPSP amplitude **(D)**, and presynaptic release (quantal content, **E**) in the absence (baseline, open) and presence (+*GluRIIA*, filled bars) of *GluRIIA* mutation. Genotypes and sample sizes are as presented in **(B)**. Mean ± SEM; *q < 0.05, **q < 0.01, ***q < 0.001, N.S. not significant; Brown-Forsythe and Welch ANOVA (non-equal variance) with Benjamini and Hochberg FDR method was used to correct for multiple comparisons.

Furthermore, we investigated the role of the vWA domain and RLR site in α2δ-3 in the long-term maintenance of PHP. PHP can be induced by genetic deletion of the postsynaptic glutamate receptor subunit, GluRIIA (Petersen et al., 1997). Since this is a genetic mutation during the lifetime of the animal, it has been used to assess the long-term maintenance of PHP. We generated double mutant animals with homozygous mutations in both *GluRIIA* and *α2δ-3* genes. The *GluRIIA* mutation alone resulted in a significant reduction in average mEPSP amplitude, accompanied by a compensatory increase in presynaptic release, indicating the induction of PHP (Figures 3A–C,E). As a result, EPSP amplitude was restored toward baseline levels in the *GluRIIA* mutant (Figures 3A,D). In contrast, in the *GluRIIA,α2δ-3* double mutant, we observed a dramatic decrease in mEPSP amplitude (q < 0.001), similar to the *α2δ-3* single mutant, with no significant change in quantal content (q = 0.30), indicating a complete block of PHP (Figures 3A–C,E).

Interestingly, when *UAS-α2δ-3^wt^* or *UAS-α2δ-3^RLR-AAA^* is expressed presynaptically in neurons in the *GluRIIA,α2δ-3* double mutant background, the quantal content is dramatically increased. This finding suggests that both the *wild-type α2δ-3* and the RLR site-mutated *α2δ-3* are capable of rescuing the long-term maintenance of PHP (Figures 3A,B,E). However, when we overexpressed *UAS-α2δ-3^DSS-AAA^* in neurons in the *GluRIIA,α2δ-3* double mutant background, there was no change in quantal content compared to the baseline value observed in the *α2δ-3^DSS-AAA^;α2δ-3* mutant alone (Figures 3A,B,E). These observations indicate that the MIDAS motif-mutated *α2δ-3* is unable to rescue PHP. Therefore, we concluded that the MIDAS motif, but not the RLR site, in α2δ-3 is required for chronic PHP. In summary, the MIDAS motif within the vWA domain is necessary for acute PHP, chronic PHP, and synaptic transmission at baseline, whereas the RLR site is only required for synaptic transmission at baseline.

### 3.5. Both the MIDAS motif and RLR site in α2δ-3 are necessary for presynaptic calcium channel abundance at baseline

**Figure 4:**
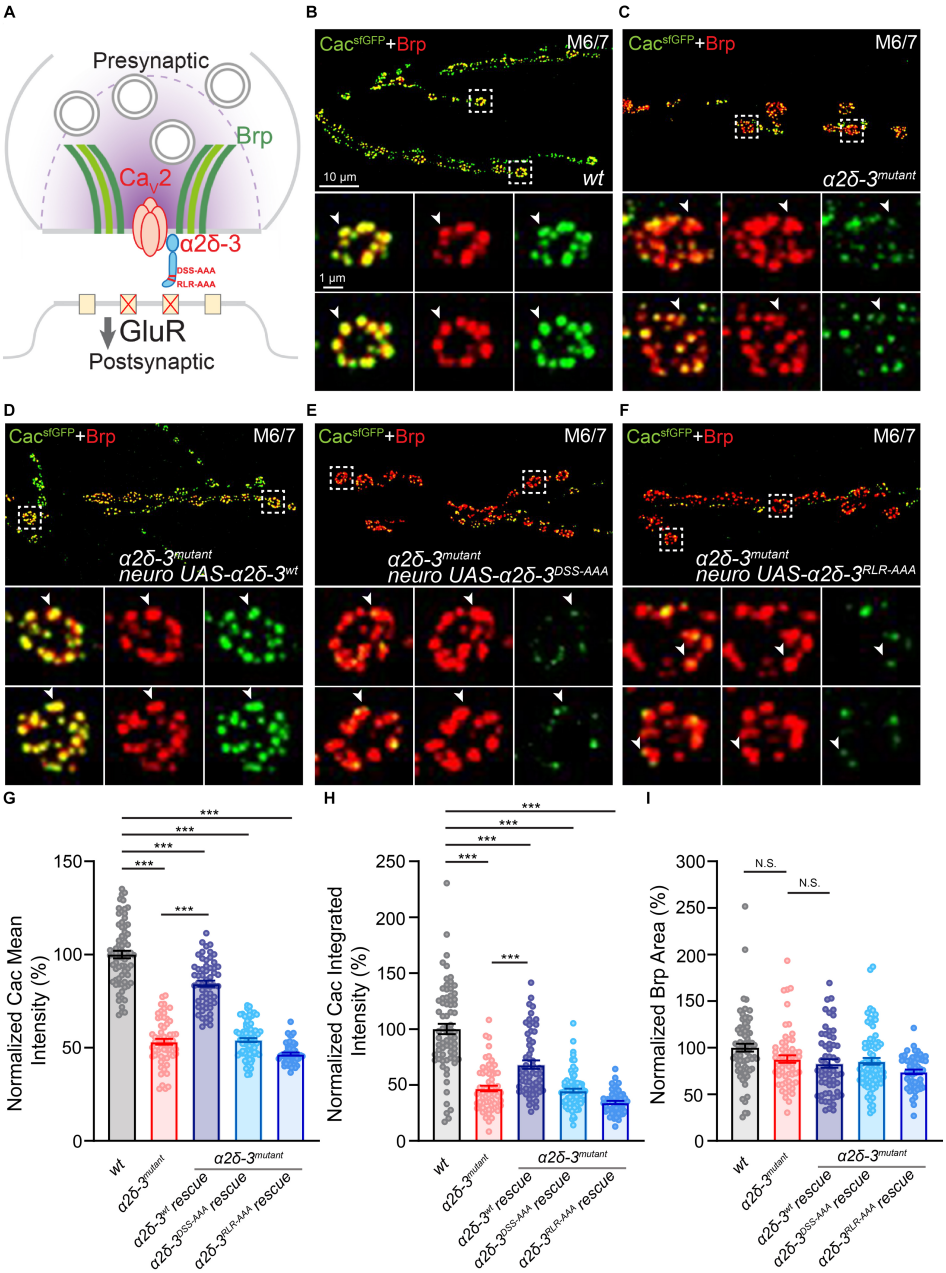
The MIDAS motif and RLR site both regulate calcium channel expression at presynaptic active zones at baseline. **(A)** Schematic to show the localization of calcium channel α1 pore-forming subunit (red) and α2δ-3 (blue, mutations are labeled in red) at presynaptic active zones (indicated by Bruchpilot, Brp, green). **(B–F)** Representative confocal images of Cac^sfGFP^ (green) and Brp (red) at the NMJ in *wild-type* (*wt*, *Cac^sfGFP^*, **B**), *α2δ-3* homozygous mutant (*α2δ-3^mutant^*, *Cac^sfGFP^;α2δ-3^k10814^/α2δ-3^106^*, **C**), motoneuron overexpression of *α2δ-3^wt^* (*α2δ-3^wt^* rescue, *Cac^sfGFP^;OK371-Gal4,α2δ-3^k10814^/α2δ-3^106^;UAS-α2δ-3^wt^*, **D**), motoneuron overexpression of *α2δ-3^DSS-AAA^* (*α2δ-3^DSS-AAA^* rescue, *Cac^sfGFP^;OK371-Gal4,α2δ-3^k10814^/α2δ-3^106^;UAS-α2δ-3^DSS-AAA^*, **E**), and motoneuron overexpression of *α2δ-3^RLR-AAA^* (*α2δ-3^RLR-AAA^* rescue, *Cac^sfGFP^;OK371-Gal4,α2δ-3^k10814^/α2δ-3^106^;UAS-α2δ-3^RLR-AAA^*, **F**) in the *α2δ-3* homozygous mutant background. Representative images of individual boutons (indicated by white boxes) are shown at higher magnification in lower panels. Individual active zones are indicated by white arrowheads. **(G–I)** Normalized average Cac^sfGFP^ mean intensity **(G)** and Cac^sfGFP^ integrated intensity **(H)** inside active zones (indicated by Brp puncta), and average Brp area **(I)** at the NMJ in *wild-type* (*wt*, *Cac^sfGFP^*, *n* = 71), *α2δ-3* homozygous mutant (*α2δ-3^mutant^*, *Cac^sfGFP^;α2δ-3^k10814^/α2δ-3^106^*, *n* = 61), motoneuron overexpression of *α2δ-3^wt^* (*α2δ-3^wt^* rescue, *Cac^sfGFP^;OK371-Gal4,α2δ-3^k10814^/α2δ-3^106^;UAS-α2δ-3^wt^*, *n* = 58), motoneuron overexpression of *α2δ-3^DSS-AAA^* (*α2δ-3^DSS-AAA^* rescue, *Cac^sfGFP^;OK371-Gal4,α2δ-3^k10814^/α2δ-3^106^;UAS-α2δ-3^DSS-AAA^*, *n* = 77), and motoneuron overexpression of *α2δ-3^RLR-AAA^* (*α2δ-3^RLR-AAA^* rescue, *Cac^sfGFP^;OK371-Gal4,α2δ-3^k10814^/α2δ-3^106^;UAS-α2δ-3^RLR-AAA^*, *n* = 52) in the *α2δ-3* homozygous mutant background. All values in the mutant and rescue groups are normalized to *wild-type* and percentage changes are shown. Mean ± SEM; *q < 0.05, **q < 0.01, ***q < 0.001, N.S. not significant; Brown-Forsythe and Welch ANOVA (non-equal variance) with Benjamini and Hochberg FDR method was used to correct for multiple comparisons.

To further investigate the roles of the MIDAS motif and RLR site in regulating calcium channel localization at presynaptic zones during PHP, we employed confocal and STED super-resolution imaging techniques (Figure 4A). Initially, we aimed to determine whether the MIDAS motif and RLR site in α2δ-3 directly influence presynaptic calcium channel abundance at baseline, utilizing confocal imaging method. In *Drosophila*, the *cacophony* (*cac*) gene encodes the α1 subunit responsible for forming the pore of Ca_v_2.1 calcium channels. To assess the synaptic expression of Cac, we used a CRISPR knock-in allele, wherein the endogenous Cac is GFP-tagged (Cac^sfGFP^; Gratz et al., 2019).

To assess Cac abundance, we performed immunolabeling and confocal imaging experiments. Cac^sfGFP^, along with the active zone component Bruchpilot (Brp; Kittel et al., 2006), was co-labeled, and the fluorescence intensity of Cac^sfGFP^ within Brp puncta was quantified. Our results revealed that synaptic Cac^sfGFP^ levels were reduced by approximately 50% in the *α2δ-3* mutant compared to the *wild-type* (Figures 4B,C,G,H). Notably, motoneuron-specific overexpression of *wild-type α2δ-3* (*UAS-α2δ-3^wt^*) resulted in a substantial increase in Cac^sfGFP^ intensity and partially restored calcium channel abundance in the *α2δ-3* mutant, approaching *wild-type* levels (*Cac^sfGFP^;α2δ-3,OK371-Gal4;UAS-α2δ-3^wt^*; Figures 4B,D,G,H). In contrast, when the MIDAS motif-mutated form of *α2δ-3* (*Cac^sfGFP^;α2δ-3^k10814^,OK371-Gal4/α2δ-3^106^;UAS-α2δ-3^DSS-AAA^*) or the RLR site-mutated form of *α2δ-3* (*Cac^sfGFP^;α2δ-3^k10814^,OK371-Gal4/α2δ-3^106^;UAS-α2δ-3^RLR-AAA^*) was expressed in motoneurons in the *α2δ-3* mutant background, calcium channel abundance remained significantly reduced compared to the *wild-type* (Figures 4B,C,E−H). These findings indicate that the mutated forms of *α2δ-3* expressed in neurons lack the ability to restore normal calcium channel distribution. Hence, the MIDAS motif and RLR site in α2δ-3 are both essential for maintaining proper calcium channel abundance at baseline.

Notably, we observed a mild but not significant reduction in the presynaptic active zone area (as indicated by Brp immunolabeling) in the *α2δ-3* homozygous mutant compared to the *wild-type* (Figures 4B,C,I). This finding suggests that the observed impairment in calcium channel abundance in the *α2δ-3* mutant is not a result of severe deficits in active zone organization. Furthermore, neuronal expression of *UAS-α2δ-3^wt^* in the *α2δ-3* mutant led to a mild reduction in active zone area, although the decrease was not statistically significant (Figure 4I). Therefore, the rescue of calcium channel abundance observed upon overexpression of *UAS-α2δ-3^wt^* is unlikely to be due to the expansion of presynaptic active zones. Collectively, these findings suggest that the disruption of the MIDAS motif or RLR site in α2δ-3 significantly reduces presynaptic calcium channel abundance, reflecting the deficits in synaptic transmission observed under these conditions using electrophysiological methods.

### 3.6. The MIDAS motif in α2δ-3 is necessary for upregulation of presynaptic calcium channel abundance during acute PHP

Recent studies utilizing super-resolution imaging have indicated that the abundance and structural organization of presynaptic scaffolding proteins and calcium channels undergo modulation during the rapid induction and long-term maintenance of PHP (Bohme et al., 2019; Gratz et al., 2019; Ghelani et al., 2023). These findings highlight the importance of presynaptic protein trafficking and stabilization in homeostatic plasticity. To further investigate the changes in presynaptic calcium channel abundance during acute PHP, we employed the STED super-resolution imaging technique. With a resolution of 40–60 nm in the X/Y dimensions, STED imaging allowed us to examine the localization of calcium channels within presynaptic active zones (approximately 200 nm in diameter). Based on our electrophysiological data suggesting the requirement of the MIDAS motif in acute PHP, we focused on understanding the role of the MIDAS motif in controlling calcium channel distribution during PHP.

**Figure 5:**
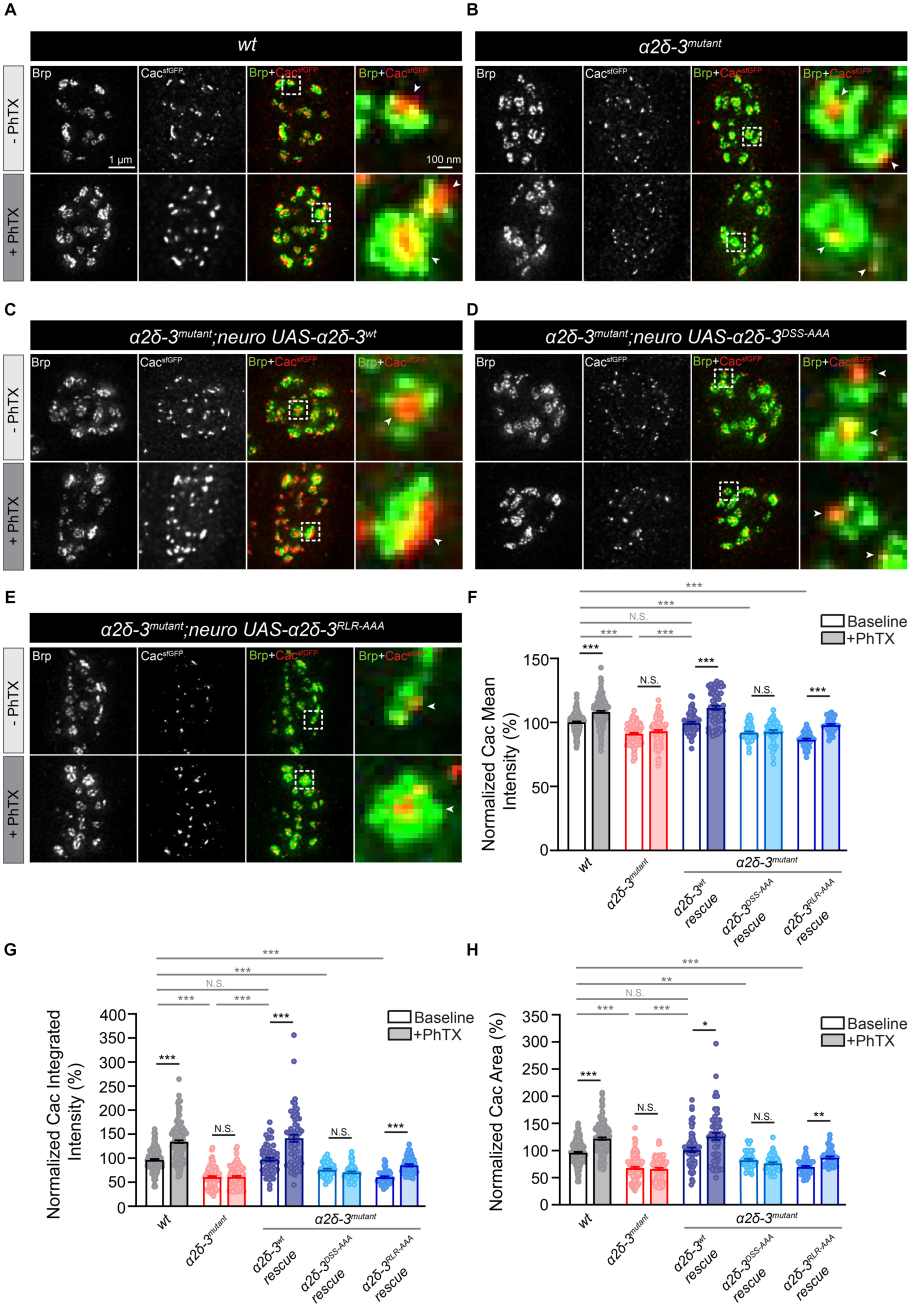
The MIDAS motif is critical for the compensatory increase in presynaptic calcium channel abundance during acute PHP. **(A–D)** Representative STED images of Cac^sfGFP^ (green) and Bruchpilot (Brp, red) at the NMJ in *wild-type* (*wt*, *Cac^sfGFP^*, **A**), *α2δ-3* homozygous mutant (*α2δ-3^mutant^*, *Cac^sfGFP^;α2δ-3^k10814^/α2δ-3^106^*, **B**), motoneuron overexpression of *α2δ-3^wt^* (*α2δ-3^wt^* rescue, *Cac^sfGFP^;OK371-Gal4,α2δ-3^k10814^/α2δ-3^106^;UAS-α2δ-3^wt^*, **C**), motoneuron overexpression of *α2δ-3^DSS-AAA^* (*α2δ-3^DSS-AAA^* rescue, *Cac^sfGFP^;OK371-Gal4,α2δ-3^k10814^/α2δ-3^106^;UAS-α2δ-3^DSS-AAA^*, **D**), and motoneuron overexpression of *α2δ-3^RLR-AAA^* (*α2δ-3^RLR-AAA^* rescue, *Cac^sfGFP^;OK371-Gal4,α2δ-3^k10814^/α2δ-3^106^;UAS-α2δ-3^RLR-AAA^*, **E**) in the *α2δ-3* homozygous mutant background in the absence (-PhTX, upper panels) and presence of PhTX (+PhTX, lower panels). Representative images of individual active zones (indicated by white boxes) are shown at higher magnification in panels on the far right. Presynaptic calcium channels localized to active zones are indicated by arrowheads. **(F–H)** Normalized average mean intensity **(F)**, integrated intensity **(G)** and area of Cac^sfGFP^ **(H)** inside each active zone (indicated by Brp) in *wild-type* (*wt*, *Cac^sfGFP^*, *n* = 157 −PhTX, *n* = 160 +PhTX), *α2δ-3* homozygous mutant (*α2δ-3^mutant^*, *Cac^sfGFP^;α2δ-3^k10814^/α2δ-3^106^*, *n* = 118 −PhTX, *n* = 104 +PhTX), motoneuron overexpression of *α2δ-3^wt^* (*α2δ-3^wt^* rescue, *Cac^sfGFP^;OK371-Gal4,α2δ-3^k10814^/α2δ-3^106^;UAS-α2δ-3^wt^*, *n* = 59 −PhTX, *n* = 59 +PhTX), motoneuron overexpression of *α2δ-3^DSS-AAA^* (*α2δ-3^DSS-AAA^* rescue, *Cac^sfGFP^;OK371-Gal4,α2δ-3^k10814^/α2δ-3^106^;UAS-α2δ-3^DSS-AAA^*, *n* = 47 −PhTX, *n* = 47 +PhTX), and motoneuron overexpression of *α2δ-3^RLR-AAA^* (*α2δ-3^RLR-AAA^* rescue, *Cac^sfGFP^;OK371-Gal4,α2δ-3^k10814^/α2δ-3^106^;UAS-α2δ-3^RLR-AAA^*, *n* = 45 −PhTX, *n* = 45 +PhTX) in the *α2δ-3* homozygous mutant background in the absence (−PhTX, upper panels) and presence of PhTX (+PhTX, lower panels). All values in the mutant and rescue groups are normalized to *wild-type* and percentage changes are shown. Mean ± SEM; *q < 0.05, **q < 0.01, ***q < 0.001, N.S. not significant; nonparametric Kruskal-Wallis test with Benjamini and Hochberg FDR method was used to correct for multiple comparisons.

To investigate this, we performed immunolabeling of Cac^sfGFP^ and Brp in five different genotypes: *wild-type*, the *α2δ-3* mutant, motoneuron overexpression of *UAS-α2δ-3^wt^* in the *α2δ-3* mutant (*Cac^sfGFP^; α2δ-3^k10814^,OK371-Gal4/α2δ-3^106^;UAS-α2δ-3^wt^*), overexpression of *UAS-α2δ-3^DSS-AAA^* in the *α2δ-3* mutant (*Cac^sfGFP^; α2δ-3^k10814^,OK371-Gal4/α2δ-3^106^;UAS-α2δ-3^DSS-AAA^*), and overexpression of *UAS-α2δ-3^RLR-AAA^* in the *α2δ-3* mutant (*Cac^sfGFP^; α2δ-3^k10814^,OK371-Gal4/α2δ-3^106^;UAS-α2δ-3^RLR-AAA^*). Immunolabeling was performed in the presence and absence of PhTX for each genotype. Upon inducing PHP by applying 20 μM PhTX for 10 min, we observed a significant increase in the mean intensity, integrated intensity, and area of Cac^sfGFP^ in the *wild-type*, indicating an upregulation of calcium channel abundance at presynaptic active zones during acute PHP (Figures 5A,F–H). However, this increase in calcium channel abundance during acute PHP was completely absent in the *α2δ-3* homozygous mutant (Figures 5B,F–H). This deficit in the upregulation of presynaptic calcium channels correlates with the impairment of the compensatory increase in neurotransmitter release observed in the *α2δ-3* mutant (Figures 2D,E,H).

We proceeded to investigate whether the trafficking of calcium channels is necessary for the observed increase in calcium channel abundance during acute PHP. Specifically, expressing *UAS-α2δ-3^wt^* in motoneurons in the *α2δ-3* mutant background led to a significant increase in the mean and integrated intensity and area of Cac^sfGFP^ upon PhTX application (Figures 5C,F–H). These findings clearly indicate the critical role of *α2δ-3* in facilitating the compensatory increase in calcium channel abundance to the presynaptic membrane during acute PHP. In contrast, overexpression of *UAS-α2δ-3^DSS-AAA^* specifically in motoneurons of the *α2δ-3* mutant did not result in any changes in calcium channel abundance upon induction of acute PHP (Figures 5D,F–H). This observation suggests that the MIDAS motif in α2δ-3 is essential for the regulation of calcium channel localization to active zones during PHP. Interestingly, when we overexpressed *UAS-α2δ-3^RLR-AAA^* specifically in motoneurons of the *α2δ-3* mutant, we observed a significant increase in the mean and integrated intensity and area of Cac^sfGFP^ following PhTX application, suggesting a rescue of calcium channel localization during PHP in the *α2δ-3^RLR-AAA^* condition (Figures 5E–H). This result indicates that the RLR site in α2δ-3 is not required for the upregulation of calcium channels during the rapid induction of PHP, consistent with our observation that the RLR site is not necessary for the increase of neurotransmitter release in PHP (Figures 2D,E,H). In summary, by using STED super-resolution imaging approach, we have provided evidence that *α2δ-3* plays a crucial role in dynamically regulating the abundance of presynaptic calcium channels during acute PHP. Furthermore, we have found that the MIDAS motif within the vWA domain, but not the RLR site, is required for the regulation of calcium channel localization in the context of acute PHP. These observations align with the essential role of the MIDAS motif in regulating presynaptic neurotransmitter release in PHP (Figures 2D,E,H).

Consistent with confocal imaging data, STED super-resolution imaging experiments revealed a significant reduction in the intensities of Cac^sfGFP^ in the *α2δ-3* mutant (Figures 5B,F–H). Furthermore, these metrics were restored to *wild-type* levels upon overexpression of *UAS-α2δ-3^wt^* in motoneurons of the *α2δ-3* mutant (Figures 5C,F–H). However, neither overexpressing *UAS-α2δ-3^DSS-AAA^* or *UAS-α2δ-3^RLR-AAA^* in motoneurons rescued the intensities of Cac^sfGFP^ in the *α2δ-3* mutant (Figures 5D–H). These results further validate the role of *α2δ-3* in regulating the abundance of presynaptic calcium channels under baseline conditions.

### 3.7. α2δ-3 is essential for upregulating presynaptic calcium channel abundance during long-term maintenance of PHP

**Figure 6:**
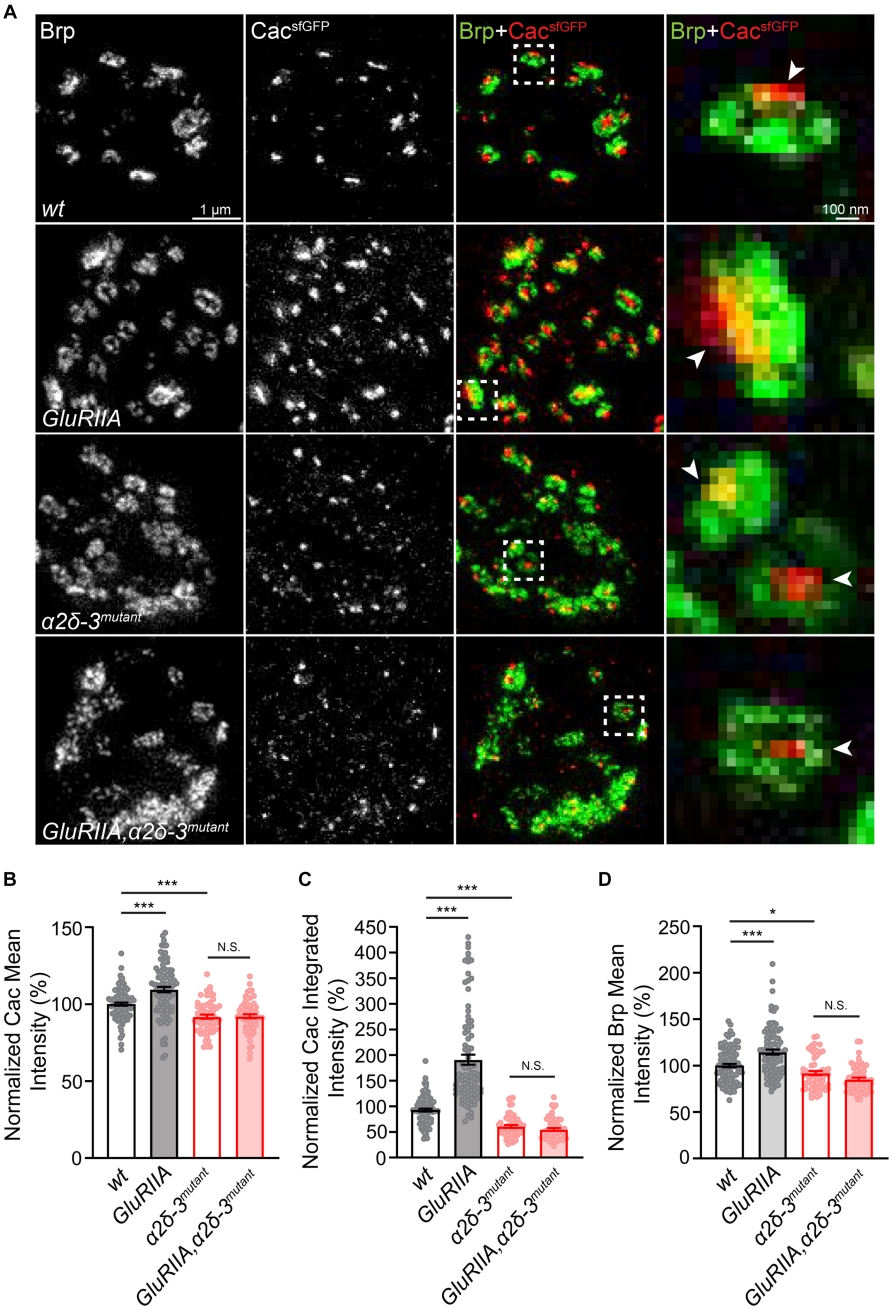
α2δ-3 is required for the increase in presynaptic calcium channel abundance in long-term maintenance of PHP. **(A)** Representative STED images of Cac^sfGFP^ (green) and Bruchpilot (Brp, red) at the NMJ in *wild-type* (*wt*, *Cac^sfGFP^*), *GluRIIA* (*GluRIIA*, *Cac^sfGFP^;GluRIIA*), *α2δ-3* mutant (*α2δ-3^mutant^*, *Cac^sfGFP^;α2δ-3^k10814^/α2δ-3^106^*), and *GluRIIA,α2δ-3* double mutant (*GluRIIA,α2δ-3^mutant^*, *Cac^sfGFP^;GluRIIA,α2δ-3^k10814^/GluRIIA,α2δ-3^106^*). Representative images of individual active zones (indicated by white boxes) are shown at higher magnification in panels on the far right. Presynaptic calcium channels are indicated by arrowheads. **(B−D)** Normalized average Cac^sfGFP^ mean intensity **(B)**, Cac^sfGFP^ integrated intensity **(C)** inside each active zone (indicated by Brp), and Brp mean intensity **(D)** in *wild-type* (*wt*, *Cac^sfGFP^*, *n* = 98), *GluRIIA* (*GluRIIA*, *Cac^sfGFP^;GluRIIA*, *n* = 95), *α2δ-3* mutant (*α2δ-3^mutant^*, *Cac^sfGFP^;α2δ-3^k10814^/α2δ-3^106^*, *n* = 59), and *GluRIIA,α2δ-3* double mutant (*GluRIIA,α2δ-3^mutant^*, *Cac^sfGFP^;GluRIIA,α2δ-3^k10814^/GluRIIA,α2δ-3^106^*, *n* = 62). All values in the mutant groups are normalized to *wild-type* and percentage changes are shown. Mean ± SEM; *q < 0.05, **q < 0.01, ***q < 0.001, N.S. not significant; nonparametric Kruskal-Wallis test with Benjamini and Hochberg FDR method was used to correct for multiple comparisons.

Finally, we aimed to determine if α2δ-3 is necessary for the compensatory increase observed in presynaptic calcium channel abundance during chronic PHP. To accomplish this, we conducted STED imaging at the NMJ and performed immunolabeling of Cac^sfGFP^ and Brp in various genetic backgrounds, including *wild-type*, *GluRIIA*, *α2δ-3*, and *GluRIIA,α2δ-3* double mutant. Our findings revealed a significant increase in presynaptic calcium channel abundance in the *GluRIIA* mutant compared to the *wild-type*, as evidenced by the elevated Cac^sfGFP^ intensities (Figures 6A–C). However, the observed increase in Cac^sfGFP^ intensities was completely abolished in the *GluRIIA,α2δ-3* double mutant compared to the *α2δ-3* mutant alone, indicating the essential role of *α2δ-3* in the compensatory increase of presynaptic calcium channels (Figures 6A–C). In line with previous findings, we also observed an elevation in the mean intensity of Brp in the *GluRIIA* mutant compared to the *wild-type*. However, this upregulation of Brp intensity was diminished in the *GluRIIA,α2δ-3* double mutant when compared to the *α2δ-3* mutant alone (Figure 6D). Collectively, these findings strongly suggest that *α2δ-3* is crucial for both the upregulation of neurotransmitter release during chronic PHP and the regulation of presynaptic calcium channels in homeostatic plasticity.

## 4. Discussion

In this study, we investigated the function of the MIDAS motif within the vWA domain and RLR site in α2δ-3 concerning the rapid induction and long-term expression of PHP (Figure 1). We provided evidence that the MIDAS motif and RLR site in *Drosophila* α2δ-3 play distinct roles in modulating calcium channel localization at presynaptic active zones, both under basal conditions and during PHP. By employing electrophysiological methods, we initially established that while the MIDAS motif and RLR site are crucial for basal synaptic transmission, only the MIDAS motif is essential for the rapid induction and sustained expression of PHP (Figures 2, 3). Intriguingly, despite disruption of the RLR site leading to deficits in basal synaptic transmission, it is not obligatory for either acute or chronic PHP. We also discovered that the reduction in basal synaptic transmission in the *α2δ-3* mutant correlates with a diminished expression of calcium channels at presynaptic active zones. Overexpression of mutated α2δ-3, either in the MIDAS motif or RLR site, was unable to restore calcium channel localization to active zones in the *α2δ-3* mutant, strongly supporting the idea that both domains are essential for calcium channel trafficking under basal conditions (Figure 4). Using STED super-resolution imaging, we showed that α2δ-3 is critical for the compensatory increase in calcium channel abundance during both acute and chronic PHP (Figures 5, 6). Moreover, we pinpointed two structural domains in α2δ-3 that are crucial for presynaptic calcium channel localization: the MIDAS motif regulates calcium channel abundance under basal conditions and during PHP, while the RLR site is only required for presynaptic localization at baseline and is dispensable for calcium channel localization during PHP. These findings highlight the pivotal role of α2δ-3 not just in controlling baseline calcium channel trafficking, but also in detecting PHP-specific signals and facilitating the synaptic activities needed for PHP.

Our previous research indicated that the loss of α2δ-3 in presynaptic motoneurons leads to a decrease in single action potential-evoked calcium influx into presynaptic terminals at baseline (Wang et al., 2016). Further, the compensatory increase in presynaptic calcium influx during PHP is entirely disrupted in the *α2δ-3* mutant. While evidence suggests that α2δ proteins regulate gating kinetics and voltage sensing of VGCCs (Shistik et al., 1995), we did not note any changes in the activation or steady-state inactivation in the *α2δ-3* mutant (Wang et al., 2016). Moreover, the RNAi-mediated reduction of the Ca_v_2.1 channel α1 subunit expression, which leads to an approximately 80% decrease in release, does not affect presynaptic homeostasis (Brusich et al., 2015). Hence, the loss of α2δ-3 entirely disrupts PHP, whereas the loss of the Ca_v_2.1 α1 subunit does not. These observations suggest that while calcium channels are essential for neurotransmitter release at baseline and during PHP, an α2δ-3-dependent and PHP-specific mechanism is required for the regulation of calcium channels during homeostatic plasticity. Our confocal and STED super-resolution imaging data strongly argue that α2δ-3 governs the trafficking and localization of presynaptic calcium channels during PHP. Moreover, we found that the MIDAS motif within the vWA domain in α2δ-3 is indispensable for increasing calcium channel abundance in PHP. Although the potential for α2δ-3 to regulate the gating and voltage-sensing activities of VGCCs during PHP remains, our data predominantly underscore the critical role of α2δ-3 in controlling calcium channel trafficking in homeostatic plasticity.

The RLR site is located at p.253–255 and the DSS site within the MIDAS motif is at p.273, 275, and 277 in the *Drosophila* α2δ-3 protein. Notably, despite being only approximately 20 amino acids apart, these two sites have distinct roles in regulating basal transmission and PHP. The MIDAS motif within the vWA domain is crucial for both basal synaptic transmission and PHP, whereas the RLR site only influences basal synaptic transmission. Their functions in controlling neurotransmitter release align well with the domain-specific impact on calcium channel localization at presynaptic active zones, both at baseline and during PHP. However, how these sites distinctly regulate calcium channel trafficking and localization in the presence or absence of perturbation remains an exciting question. The cryo-EM structure of the human Ca_v_1.2/ Ca_v_β3/ Ca_v_α2δ-1 protein complex implies that α2δ proteins supersede chaperone proteins that retain the α1 pore-forming subunit of VGCCs in the ER (Chen et al., 2023a). This interaction between α2δ and the α subunit directly facilitates the assembly and forward trafficking of calcium channels.

Contrarily, gabapentin’s binding to α2δ-1 and α2δ-2 hinders the recycling of calcium channels from intracellular recycling endosomes (Tran-Van-Minh and Dolphin, 2010). Intriguingly, mammalian α2δ-3 does not directly bind gabapentin (Marais et al., 2001). However, in *Drosophila* α2δ-3, three proximate residues—tyrosine (p.250), arginine (p.255), and tryptophan (p.257) located in or around the RLR site and directly within the hydrophobic binding pocket are conserved in comparison to human α2δ-1 (Chen et al., 2023b). These three residues are crucial for hydrogen bond interactions with L-leucine and gabapentin in human α2δ-1, suggesting the potential for endogenous molecules to interact with α2δ-3 via the RLR site or its proximate residues to control calcium channel trafficking.

While we cannot completely rule out that mutations in the MIDAS motif might influence protein expression levels or degradation kinetics, our findings suggest that the MIDAS motif and the RLR site in α2δ-3 may regulate calcium channel trafficking through distinct intracellular pathways. Identifying novel binding partners of α2δ that influence VGCC trafficking will broaden our knowledge of the molecular mechanisms underpinning α2δ-dependent regulation of presynaptic calcium channels (Kadurin et al., 2017). Moreover, the disruption of postsynaptic glutamate receptors could potentially stimulate presynaptic signaling pathways, promoting MIDAS motif-mediated trafficking of calcium channels to active zones. The means by which intracellular organelles and molecules essential for channel forward trafficking and turnover sense and adapt to neuronal activity changes remains a fascinating question (Jarvis and Zamponi, 2007; Wang et al., 2012; Cunningham et al., 2022). Unraveling how the interaction between α2δ-3 and its binding partners regulates calcium channel trafficking during PHP is a crucial direction for future investigations.

In a previous study, *α2δ-3* homozygous null mutant embryos showed a deficit in bouton formation (Kurshan et al., 2009). Intriguingly, this same research observed that the intensity of individual Brp puncta was largely consistent between the *α2δ-3* null mutant and the *wild-type* control in 21 h after-egg-laying (AEL) embryos. A separate study indicated a 20% reduction in Brp puncta number in a strong loss-of-function allele of *α2δ-3* during the 3^rd^ instar larval stage (Dickman et al., 2008). Such findings suggest that while there are impairments in Brp localization in the *α2δ-3* mutant, these disruptions are notably less pronounced than the deficits observed in calcium channel abundance or synaptic transmission. In our own research, we observed a modest reduction in Brp area (Figure 4I) and the intensity of individual Brp puncta (Figure 6D), which aligns with the previous reports.

In conclusion, our study provides evidence that α2δ-3 has a critical role in controlling presynaptic calcium influx by regulating the trafficking and expression of calcium channels at presynaptic active zones. However, these findings do not rule out the potential for α2δ-3 to modulate neurotransmitter release through mechanisms independent of VGCCs. Previously, we showed that the size of the EGTA-sensitive vesicle pool significantly decreases when α2δ-3 is disrupted. Moreover, we discovered that α2δ-3 has a genetic interaction with the synaptic scaffolding protein Rim and plays a role in regulating the readily releasable vesicle pool during PHP (Wang et al., 2016). As such, we propose that the function of α2δ-3 may extend beyond the regulation of calcium channel trafficking. The multifaceted role of α2δ-3 in PHP highlights its fundamental function in integrating and coordinating various signaling pathways for the precise modulation of neurotransmitter release in homeostatic plasticity.

## Data availability statement

The original contributions presented in the study are publicly available. This data can be found here: https://github.com/wanglab-georgetown/alphafold_a2d3.

## Ethics statement

The manuscript presents research on animals that do not require ethical approval for their study.

## Author contributions

YZ: Data curation, Formal analysis, Investigation, Writing – review & editing. TingW: Data curation, Formal analysis, Investigation, Writing – review & editing, Methodology, Visualization. YC: Formal analysis, Investigation, Visualization, Writing – review & editing, Data curation. TC: Formal analysis, Investigation, Methodology, Software, Visualization, Writing – review & editing, Data curation. MK: Investigation, Writing – review & editing. SV: Writing – review & editing. TingtW: Data curation, Formal analysis, Investigation, Methodology, Visualization, Writing – review & editing, Conceptualization, Funding acquisition, Project administration, Resources, Software, Supervision, Writing – original draft.
