# Corrigendum: Role of α2δ-3 in regulating calcium channel localization at presynaptic active zones during homeostatic plasticity

**DOI:** 10.3389/fnmol.2023.1344096

**Published:** 2023-12-07

**Authors:** Yanfeng Zhang, Ting Wang, Yimei Cai, Tao Cui, Michelle Kuah, Stefano Vicini, Tingting Wang

**Affiliations:** ^1^Department of Pediatric Neurology, First Hospital of Jilin University, Changchun, Jilin, China; ^2^Department of Pharmacology and Physiology, Georgetown University Medical Center, Washington, DC, United States; ^3^Interdisciplinary Program in Neuroscience, Georgetown University Medical Center, Washington, DC, United States

**Keywords:** α2δ-3, voltage gated calcium channel, presynaptic homeostatic plasticity, neurotransmitter release, trafficking, autism, epilepsy, gabapentin

In the published article, there was an error in the affiliations. Instead of “1 Department of Pharmacology and Physiology, Georgetown University Medical Center, Washington, DC, United States; 2 Interdisciplinary Program in Neuroscience, Georgetown University Medical Center, Washington, DC, United States; PRESENT ADDRESS Yanfeng Zhang, Department of Pediatric Neurology, First Hospital of Jilin University, Changchun, Jilin, China” it should be “1 Department of Pediatric Neurology, First Hospital of Jilin University, Changchun, Jilin, China; 2 Department of Pharmacology and Physiology, Georgetown University Medical Center, Washington, DC, United States; 3 Interdisciplinary Program in Neuroscience, Georgetown University Medical Center, Washington, DC, United States”.

The authors apologize for this error and state that this does not change the scientific conclusions of the article in any way. The original article has been updated.

